# Species Diversity and Distribution of Non-volant Small Mammal between Restoration, Boundary, Disturbed and Undisturbed Area in Cameron Highlands, Malaysia

**DOI:** 10.21315/tlsr2023.34.1.10

**Published:** 2023-03-31

**Authors:** Nur Syakirah Baharudin, Marina Mohd. Top@Mohd. Tah, Syaizwan Zahmir Zulkifli, Nurul Izza Ab Ghani, Hafidzi Mohd Noor, Nabilah Hamidah Sabar@Sabal

**Affiliations:** 1 Department of Biology, Faculty of Science, Universiti Putra Malaysia, 43400 UPM Serdang, Selangor, Malaysia; 2 Center of Foundation Studies for Agricultural Science, Universiti Putra Malaysia, 43400 UPM Serdang, Selangor, Malaysia; 3 Department of Plant Protection, Faculty of Agriculture, Universiti Putra Malaysia, 43400 UPM Serdang, Selangor, Malaysia; 4 Forest Department of Peninsular Malaysia, Jalan Sultan Salahuddin, 50660 Kuala Lumpur, Malaysia

**Keywords:** Inventory, Richness, Forest Reserve, Rodentia, Camera Trap, Inventori, Kekayaan, Hutan Simpan, Rodensia, Perangkap Kamera

## Abstract

Deforestation in Cameron Highlands, Malaysia has increased significantly in the past few years to accommodate the growing population of Cameron Highlands. This led to a rapid urbanisation in Cameron Highlands which increased anthropogenic activities, causing degradation of the natural environment. Such environmental changes highlight the necessity of wildlife and resource inventories of available forested areas to improve existing conservation and management plans, especially for threatened taxa such as the non-volant small mammals. However, very few studies are known to focus on the effect of deforestation on non-volant small mammals, especially in the adjacent forest. This survey aimed to document non-volant small mammals from four habitat types (restoration areas, boundary, disturbed and undisturbed areas) of Terla A and Bertam, and undisturbed forest of Bukit Bujang Forest Reserve, Cameron Highlands, Malaysia. Samplings were conducted in two phases between August 2020 to January 2021. A total of 80 live traps were deployed along the transect lines in all three study sites, and 10 camera traps were set randomly in each forested area. Results demonstrated that species diversity (*H′*) is higher at Terla A Forest Reserve compared to Bertam and Bukit Bujang Forest Reserve. In contrast, species diversity in the boundary area (*S* = 8, *H′* = 2.025) and disturbed forest area (*S* = 8, *H′* = 1.992) had similar number of species (*S*) compared to others study habitat; restoration area had the lowest species diversity (*S* = 3, *H′* = 0.950). *Berylmys bowersi* was the most captured species from trappings and *Lariscus insignis* was the most frequently recorded species from camera trappings for all study sites. The results of the survey provided new information on non-volant small mammals in Cameron Highlands for future research, conservation, and management.

## Abstract (fr)

Penebangan hutan di Cameron Highlands, Malaysia telah meningkat dengan ketara dalam beberapa tahun kebelakangan ini bagi menampung populasi penduduk di Cameron Highlands yang semakin meningkat. Ini membawa kepada urbanisasi pesat di Cameron Highlands yang meningkatkan aktiviti antropogenik, menyebabkan kemerosotan alam sekitar semula jadi. Perubahan alam sekitar sebegini menyerlahkan keperluan hidupan liar dan inventori sumber kawasan hutan yang ada untuk menambah baik rancangan pemuliharaan dan pengurusan sedia ada, terutamanya untuk taxa yang terancam seperti mamalia kecil yang tidak terbang. Walau bagaimanapun, sangat sedikit kajian yang diketahui memberi tumpuan kepada kesan penebangan hutan ke atas mamalia kecil yang tidak terbang, terutamanya di hutan bersebelahan. Tinjauan ini bertujuan untuk mendokumentasikan mamalia kecil tidak terbang daripada empat jenis habitat (kawasan pemulihan, sempadan, kawasan terganggu dan tidak terganggu) Terla A dan Bertam, dan hutan tidak terganggu Hutan Simpan Bukit Bujang, Cameron Highlands, Malaysia. Persampelan telah dijalankan dalam dua fasa antara Ogos 2020 hingga Januari 2021. Sebanyak 80 perangkap hidup telah digunakan di sepanjang garisan *transect* di ketiga-tiga tapak kajian, dan 10 perangkap kamera ditetapkan secara rawak di setiap kawasan hutan. Keputusan menunjukkan bahawa kepelbagaian spesies (H*′*) adalah lebih tinggi di Hutan Simpan Terla A berbanding Hutan Simpan Bertam dan Bukit Bujang. Sebaliknya, kepelbagaian spesies di kawasan sempadan (S = 8, H′ = 2.025) dan kawasan hutan terganggu (S = 8, H′ = 1.992) mempunyai bilangan spesies (S) yang sama berbanding habitat kajian yang lain; kawasan pemulihan mempunyai kepelbagaian spesies yang paling rendah (S = 3, H′ = 0.950). *Berylmys bowersi* adalah spesies yang paling banyak ditangkap daripada perangkap dan *Lariscus insignis* adalah spesies yang paling kerap direkodkan daripada perangkap kamera untuk semua tapak kajian. Hasil tinjauan memberikan maklumat baharu tentang mamalia kecil yang tidak terbang di Cameron Highlands untuk penyelidikan, pemuliharaan dan pengurusan masa hadapan.

**Highlights**
- Most number of non-volant small mammals discovered belonged to the family Muridae.
- Listed as Critically Endangered by IUCN Red List, *Manis javanica* was recorded at the undisturbed forest of Bukit Bujang, Cameron Highlands.
- *Tragulus kanchil* is a shy species with new records in Cameron Highlands.

## INTRODUCTION

Malaysia is one of the biodiversity hotspots in the tropical region of Southeast Asia, with a high faunal diversity including small mammals. Small mammals are described as any mammals that weigh less than 5 kg (Lim & Pacheco 2016) and are highly diverse with a range of tolerance to habitat disturbance (Rickart *et al.* 1991). The diversity of mammals within the Malaysian territory is quite significant with at least 440 species of mammals recorded (Department of Wildlife and National Parks [DWNP] 2016), of which 15% (66 species) are endemic to Malaysia (Dee *et al.* 2019).

According to Chan (2019), Cameron Highlands hosts more than 700 plant species, where 145 of them are endemic including 32 orchid species. A total of 56 mammals, 199 birds, 58 reptile and 14 amphibian species have also been recorded here. From this list, the pitcher plant, Serow and Mountain Peacock-Pheasant are listed in the 2021 IUCN Red List of Threatened Species.

For years, the degradation of nature due to anthropological development of highland areas in Malaysia has been alarming. The foggy climate, cool temperatures and beautiful scenery from the top of the hills have inspired tourism related developments in the highlands. The construction of roads and resorts has changed the habitat for species that inhabit the mountainous area. According to Weebers and Idris (2016), Cameron Highlands was developed for sanatorium and amusement purpose, and also as an agricultural site with tea and vegetables farm. Indiscriminate land clearing for farming and agriculture has created untold damage and pollution to its once pristine environment. Unsustainable agriculture has severe impacts on the environment, wildlife and tourism of Cameron Highlands (Barrow *et al.* 2005; Razali *et al.* 2018). The different types of plant present at different habitats may help in identifying the species of non-volant small mammals (Haziq *et al.* 2021).

Cameron Highlands is rich in diversity of faunas including insects (54 and 59 species from the Order Coleoptera and Lepidoptera, respectively), 13 species of bats and five species of non-volant small mammals (Shahfiz *et al.* 2011; Abdullah *et al.* 2011; Nur Amira *et al.* 2017). Unfortunately, according to Abdullah *et al.* (2011) and Palanivel (as cited in *The Star Online* 2013), due to high demand for the tourism industry, Cameron Highlands has been threatened with degradation of nature over the years. According to Siti Salwa (2020), illegal land-clearing and farming activities have been blamed for the landslides, mudslides and floods that frequently occur in Cameron Highlands. The environmental changes highlight the necessity of wildlife and resource inventories at available forested areas to help in existing conservation and management plans, especially for threatened taxa such as the non-volant small mammals. Consequently, these mammals may be more sensitive to forest loss resulting in the mammals avoiding disturbed and open habitats (Kingston *et al.* 2003). Due to the dependence on forest, these species were probably adversely affected by deforestation and other forest disturbances (Lane *et al.* 2006; Struebig *et al*. 2008). However, there are very few studies that have focused on the effect of deforestation on non-volant small mammals especially in the adjacent forest.

The main purpose of this study is to determine species diversity and distribution of non–volant small mammals in different habitats representing restoration or rehabilitated, edge or boundary, disturbed forest and undisturbed forest area. Factors that may influence the observed differences can be possibly identified by comparing the diversity and distribution of non-volant small mammals in the respective habitat types. Basic information on species diversity is essential for forest management and local authorities to develop sound management plans. However, a lack of basic knowledge on biodiversity could lead to non-holistic local planning and would have a negative impact on the environment, especially on fauna diversity.

## MATERIALS AND METHODS

### Study Site

The study areas were classified into four habitats, namely restoration, boundary, disturbed forest and undisturbed forest area. According to Butler and Lawrence (2019), the categorisation of forest sites was done based on the frequency of human activities in the surrounding areas, distance to human settlement or activities, types of forest, and the status of the forest (protected or non-protected). The presence of various types of habitats plays a major role in the richness of Malaysia’s diversity (Dee *et al.* 2019).

This study was conducted in Terla A Forest Reserve (04°35′36.6″ N, 101°22′54.7″ E) with an elevation of 1300 m–1500 m. Genus *Casuarina* (*rhu bukit*) dominated the restoration area. The average diameter and plant height in the restoration area were 10 mm and 0.90 m, respectively. Flowering plants such as genus *Physalis* (*buah letup*), wild orchids, *Lagenaria siceraria* (bottle guard) and genus *Nepenthes* (pitcher plant) were seen growing wildly near the hill boundaries. The forest is overgrown with Dipterocarp trees, grasses and herbaceous plants. Minimal human disturbance was observed with the presence of a piping line that provide water source to the agricultural farm nearby. The distance from the restoration area and forest area were approximately 5 m apart.

**Figure 1:**
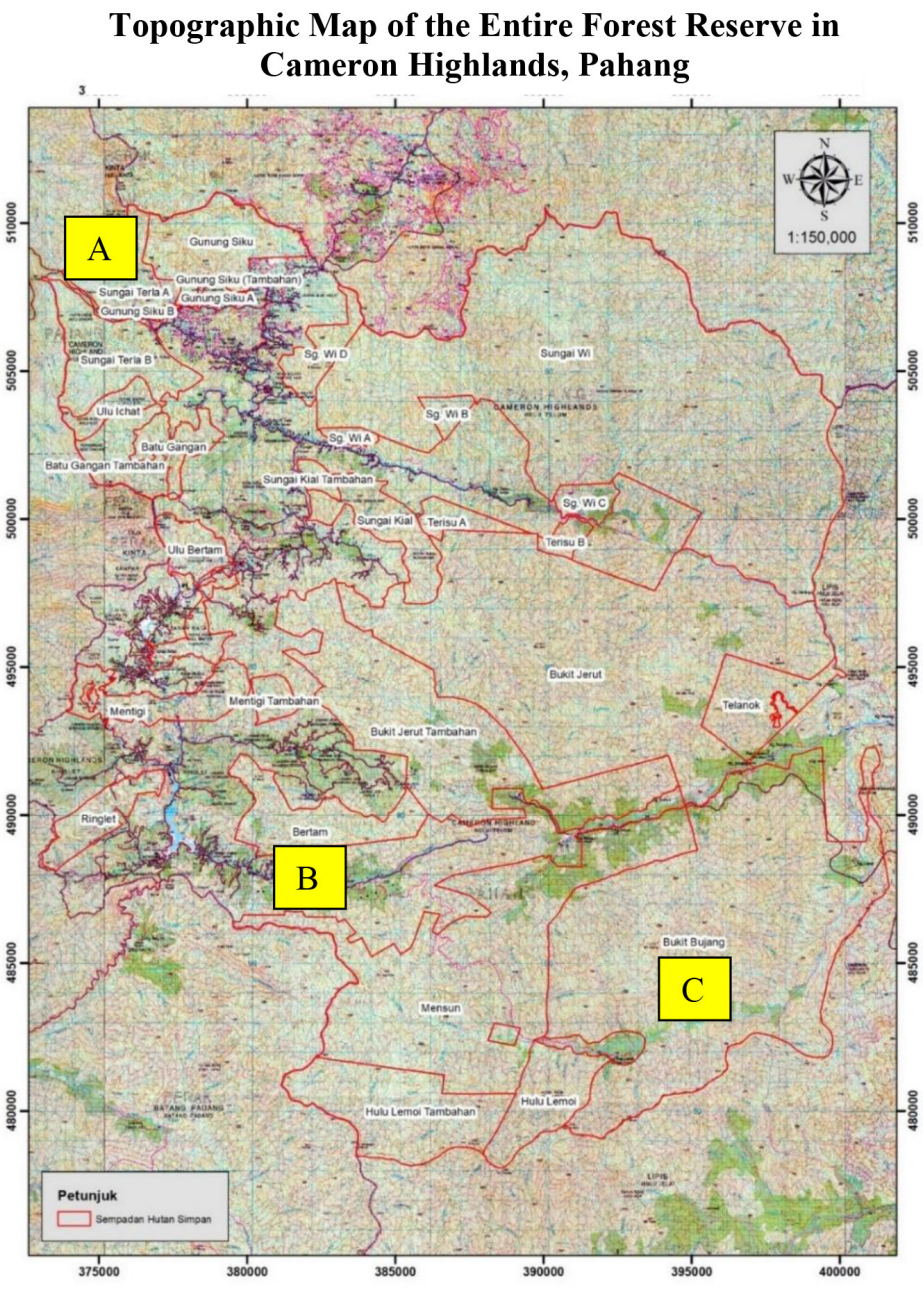
Location of (A) Terla FR A-1 (04°35′36.6″ N, 101°22′54.7″ E), (B) Bertam FR-3 (04°25′15.0″ N, 101°26′ 41.4″ E) and (C) Bukit Bujang FR (04°24′ 07.06″ N, 101°35′37.28″ E) Cameron Highlands, Pahang (Adapted from Forestry Department of Peninsular Malaysia 2020).

Bertam Forest Reserve (04°25′ 15.0″ N, 101°26′ 41.4″ E) has an elevation of 1200 m–1300 m in Cameron Highlands, Pahang (Fig. 1). The restoration trees were dominated by genus *Casuarina* (*rhu bukit*). Flowering plants such as *Morus* (mulberry tree), *Mentha* (mint plant) and *Bambusa* (bamboo tree) were seen growing wildly near the hill boundaries. The average diameter and plant height in the restoration area were 10 mm and 0.90 m, respectively. The forest area has a small river as a source of water and is dominated by *Eugeissona* (*bertam*) and *Musa balbisiana* (sweet wild banana).

The study was also conducted at Bukit Bujang Forest Reserve (04°24′ 07.06″ N, 101°35′37.28″ E) which has an elevation of 400 m–500 m to compare the state of the diversity of non-volant small mammals in two different areas namely the Disturbed Forest Area and Undisturbed Forest Area (Fig. 1). Vegetation for Bukit Bujang Reserve Forest has been dominated by trees of 5 m–10 m high. Different type of trees such as genus *Eugeissona* (*bertam*), genus *Calamus* (*rotan*), genus *Licuala* (*palas*) and genus *Oncosperma* (*bayas*) dominated this forest. This area has sufficient water source from rivers such as the Lemoi River.

Terla A and Bertam Forest Reserved are restoration areas listed under “Restoration, Rehabilitation, and Reclamation (3RSM)” programme in Cameron Highlands. Cameron Highlands is one of the areas affected by floods in 2014. Therefore, Forestry Department of Peninsular Malaysia initiated a restoration programme in the Eleventh Malaysia Plan (2016–2020) with an estimated area of 1640 ha in works. Almost all the undisturbed forests nearby in Cameron Highlands are located at low altitudes (Forestry Department of Peninsular Malaysia 2020).

Restoration of an area is defined as any intentional activity that initiates or accelerates the recovery of an ecosystem from a degraded state (http://www.ipbes.net/). Forest boundary is particularly a transition area between forest and non-forest where a dense forest is gradually opening up to tree-free land, as for example at the timber line or at the boundary of deserts (Kleinn 2007). Forest areas disturbed by human activities in the past may naturally regain features that characterize primary forests while others that don’t will require restoration (Lindenmayer *et al.* 2014).

The survey was based on two phases of sampling between August 2020 to January 2021. The sampling periods included the dry season and the wet season for Cameron Highlands. Sampling and monitoring non-volant small mammals were replicated twice for all three study sites to minimise bias in collecting data.

### Sampling Method

In this study, two methods were used, i.e., live trapping and camera trapping that target the more typical terrestrial non-volant small mammals. Advancements in camera-trapping technology have led to the widespread use of this survey method in the study of terrestrial mammals (Wearn & Glover-Kapfer 2017; Jessica *et al.* 2021). The two methods were used for a more comprehensive inventory documentation of species such as using camera traps for the documentation of less trappable species (Tasker & Dickman 2001; De Bondi *et al.* 2010; Thomas *et al.* 2020). It also allowed for a direct comparison between live trapping and camera trapping efforts in the same location and was aimed to increase the chances of recording targeted samples of non-volant small mammals in the study area (Francesco *et al.* 2010).

### Live Trap

According to Hoffman *et al.* (2010), the most suitable and easiest trapping method for small mammals is to place traps at more or less fixed intervals at parallel and equidistant transect lines along an equal intervals line, which can cover all types of habitats.

In this study, the transect line constructed was different for each habitat where the boundary area had a longer transect line of 750 m and 250 m at restoration, disturbed forest and undisturbed forest. Each transect line was placed at least 50 m apart, which could be adjusted based on the terrain, accessibility and vegetation types (Pearson & Ruggiero 2003).

**Figure 2:**
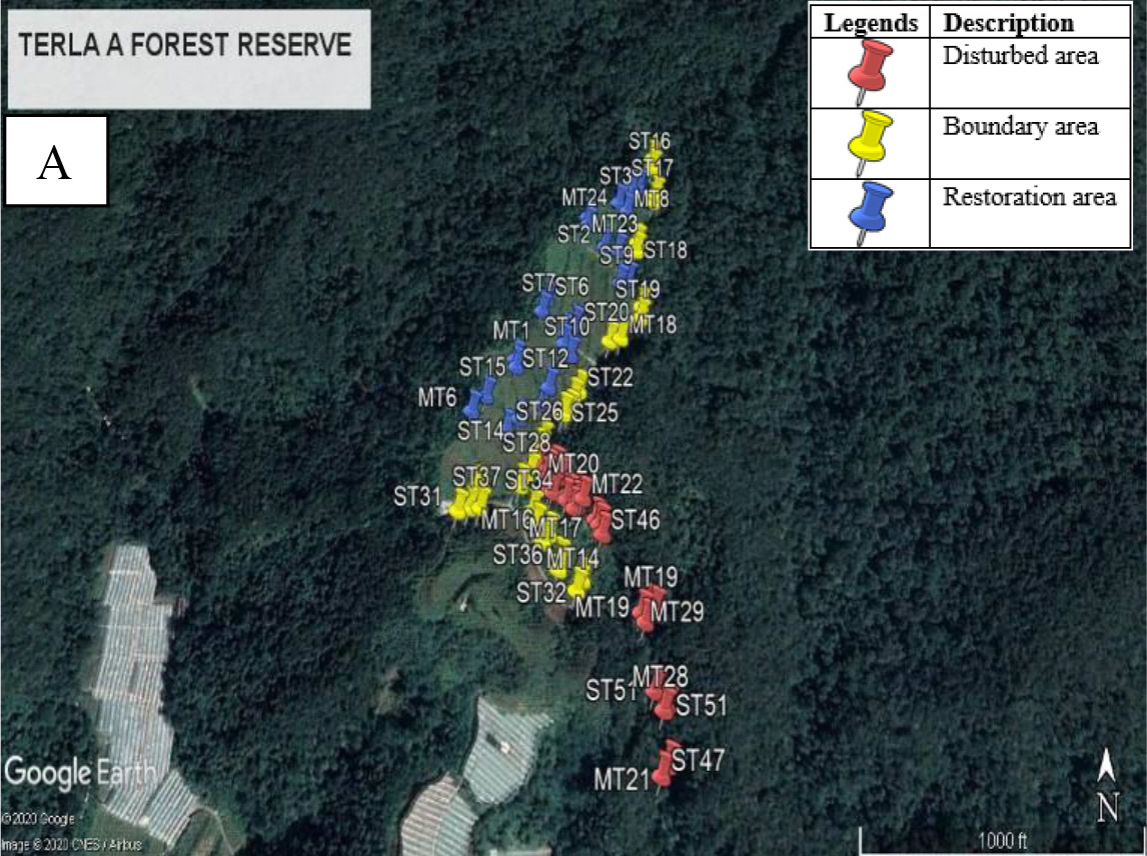

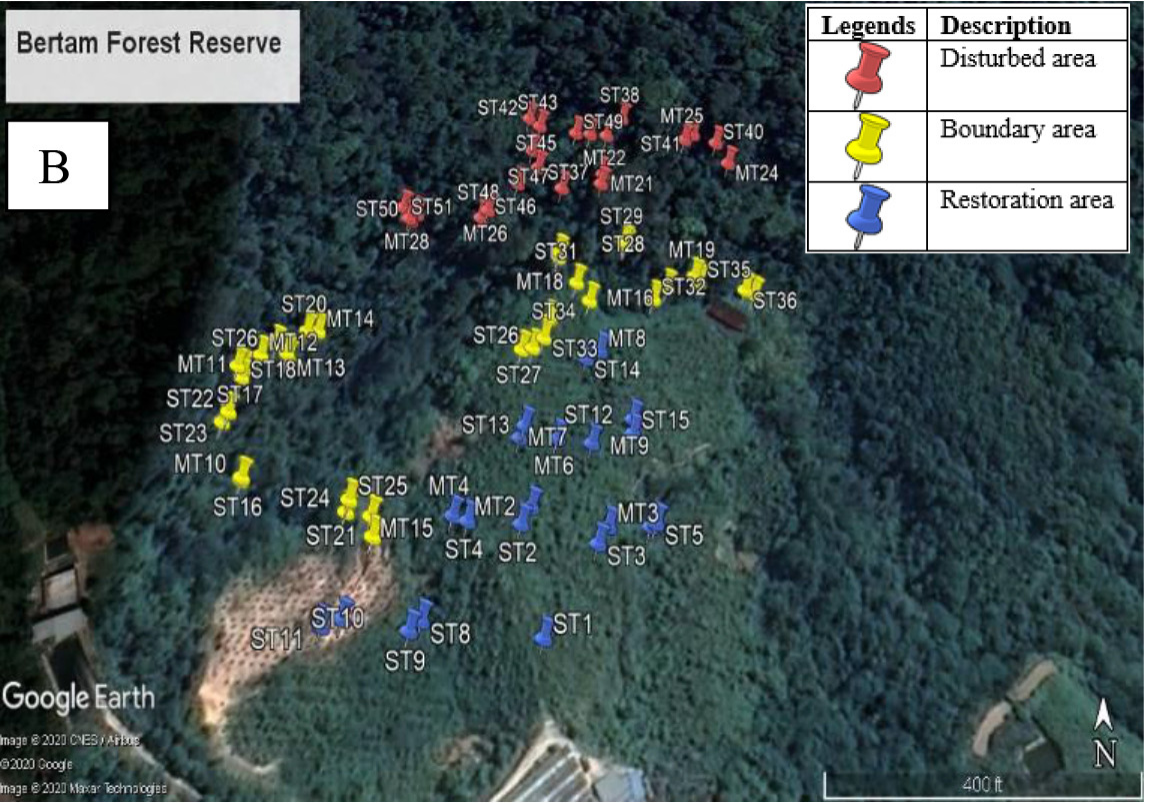

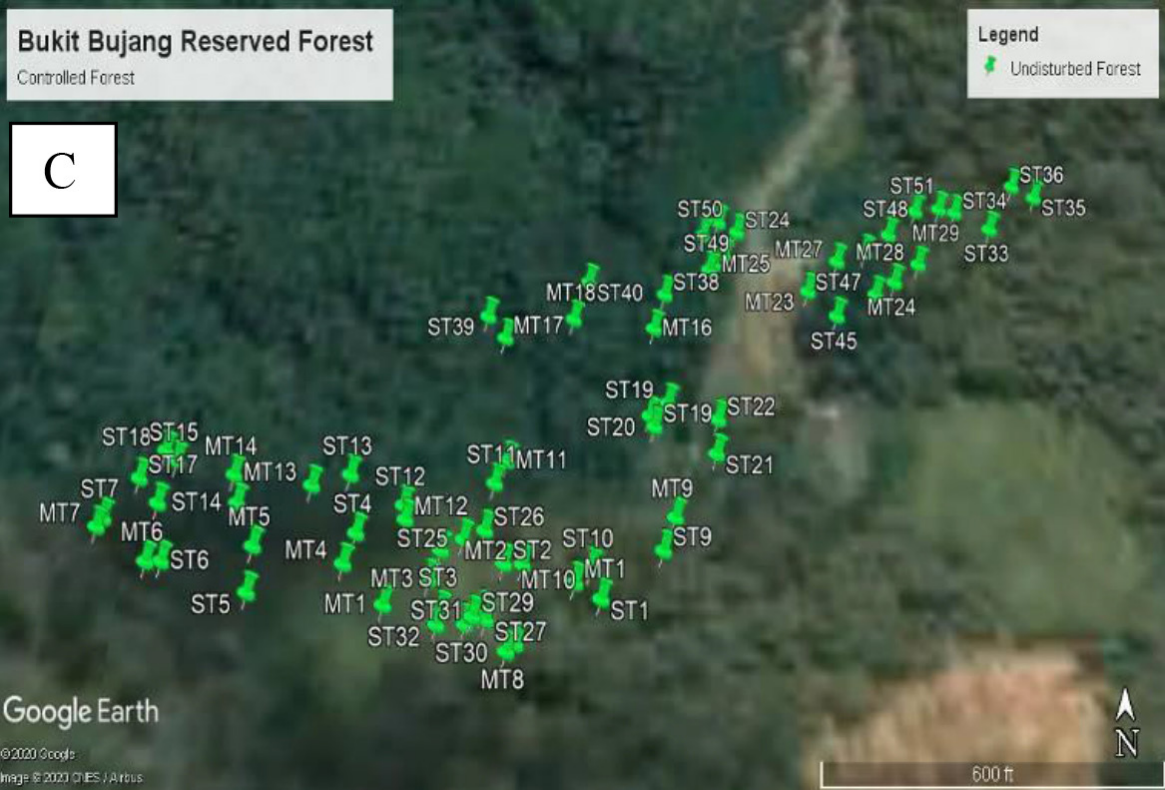
Distribution of 80 duplicated live traps in each study site: (A) Terla FR A-1, (B) Bertam FR-3 and (C) Bukit Bujang FR (Adapted from Google Earth Pro V 7.1).

A distribution of 80 units of wire mesh live traps measuring 25 cm × 11.5 cm × 12 cm (small traps) and 81 cm × 38 cm × 25 cm (medium traps) were deployed along the transect line in each study site based on ecological conditions and topography of the surrounding area (Figs. 2A, 2B and 2C). Live traps were also placed on dead logs, semi-open areas that resemble animal trails, near thistle plants and branches of tree (Jambaari *et al.* 1999; Zakaria *et al.* 2001; Norfahiah *et al.* 2012) to increase trapping success likelihood. The distances between traps were 10 m and were each setup at 50 m interval points. The surface of the traps was covered with forest litters to provide thermal insulation for the captured sample and also acting as camouflage (Torre *et al.* 2004). Six types of baits were equally distributed among the habitats to compare the effectiveness in luring small mammals. Live traps were baited alternately with sweet potato, banana, palm oil fruits, chicken meat left-overs, roasted prawn and salted fish set for five consecutive days and four consecutive nights. Different types of baits were used to increase the number of species attracted. According to Kok *et al.* (2013) baits are commonly used for surveying small mammal communities, not only because they attract large numbers of these mammals, but also because they provide sustenance for trapped individuals.

Any captured non-volant small mammals were transferred into dark plastic bags (Payne *et al.* 2008). Cotton wools coated with chloroform were prepared to anesthetize the samples (Barnett & Dutton 1997). Anesthetized captured samples were then measured (weight, head and body length, tail length, ear length, hindfoot length), sexed and species identification was done in reference to Phillipps and Phillipps (2016) and Francis (2019). All non-volant small mammals captured were tagged on the nail using nail polish (Tingga *et al.* 2021) for identification and then released back to their habitat (Shukor 2001).

Correct identification of species is of primary importance to many studies (Chaval *et al.* 2010). According to Pimsai *et al.* (2014) it incorporates a detailed summary of descriptive characters of the external, cranial and dental morphology (i.e., colour patterns, shape of body or head, size) and measurements (i.e., head and body length, tail length, ear length, hindfoot length) for each of the non-volant small mammal species.

### Camera Trap

Camera trapping is an established method for the monitoring of medium- to large-sized mammals populations (Garden *et al.* 2007; Trolle *et al.* 2008; Mccleery *et al*. 2014; De Bondi *et al.* 2010), but are rarely used for smaller mammals. However, according to Jackson *et al.* (2006) camera traps have been used to record a wide range of fauna in various habitats. Camera trapping is a non-invasive method that generally causes minimum disturbance to the target species, can be left unattended for several days, and are ideally suited for studying rare, elusive, and nocturnal/crepuscular animals that avoid humans (Francesco *et al.* 2010). The big advantage of using camera traps is that it provides real time record of the animal presence. According to Francesco *et al.* (2010), camera trapping provides information on activity patterns (from the date and time recorded in the image), behaviour and pelage characteristics that enable individual identification.

Ten camera trap brands from three different models, Digital Trail Camera (HC-800 A), Hunting Trail Camera and Wildlife Camera were used in this study. These cameras used infrared camera sensors triggered by heat and motion set at 1-second interval between exposures unless the animal was running at high speed. We programmed five camera traps to record three photographs for every trigger and another five camera traps with 15-second video duration. The three photos were subjectively defined as a single photographic “event.” These settings were selected to provide photographs of the individual in different positions and ultimately increase identification accuracy (Thomas *et al.* 2020). The cameras were installed on suitable trees with a height of 30 cm–50 cm from the ground at optimum angles overlooking the animal trails without the camera view being blocked by any objects. We identified non-volant small mammal species based on several identification guides (Francis & Barrett 2008; Medway 1978).

**Figure 3:**
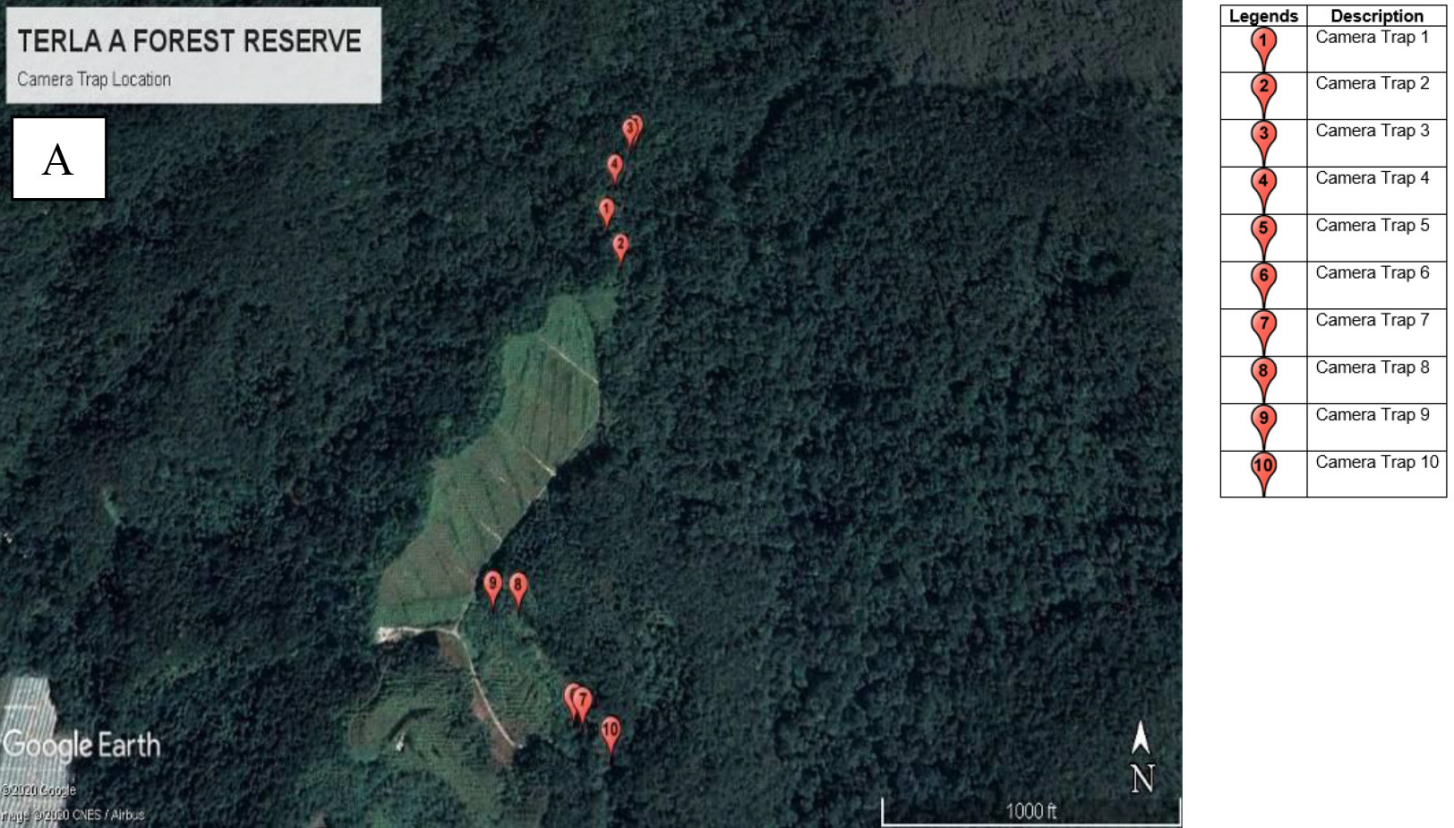

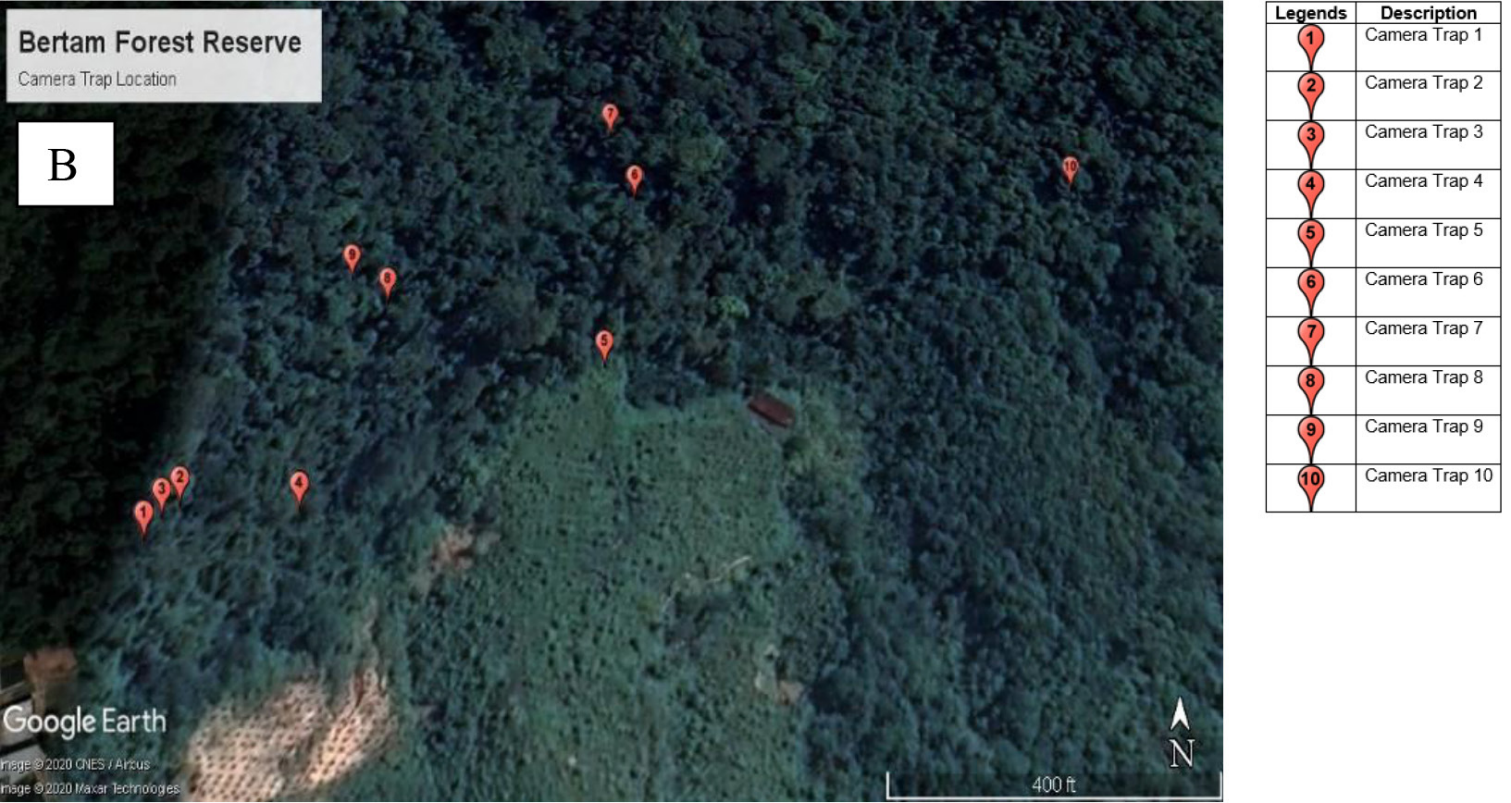

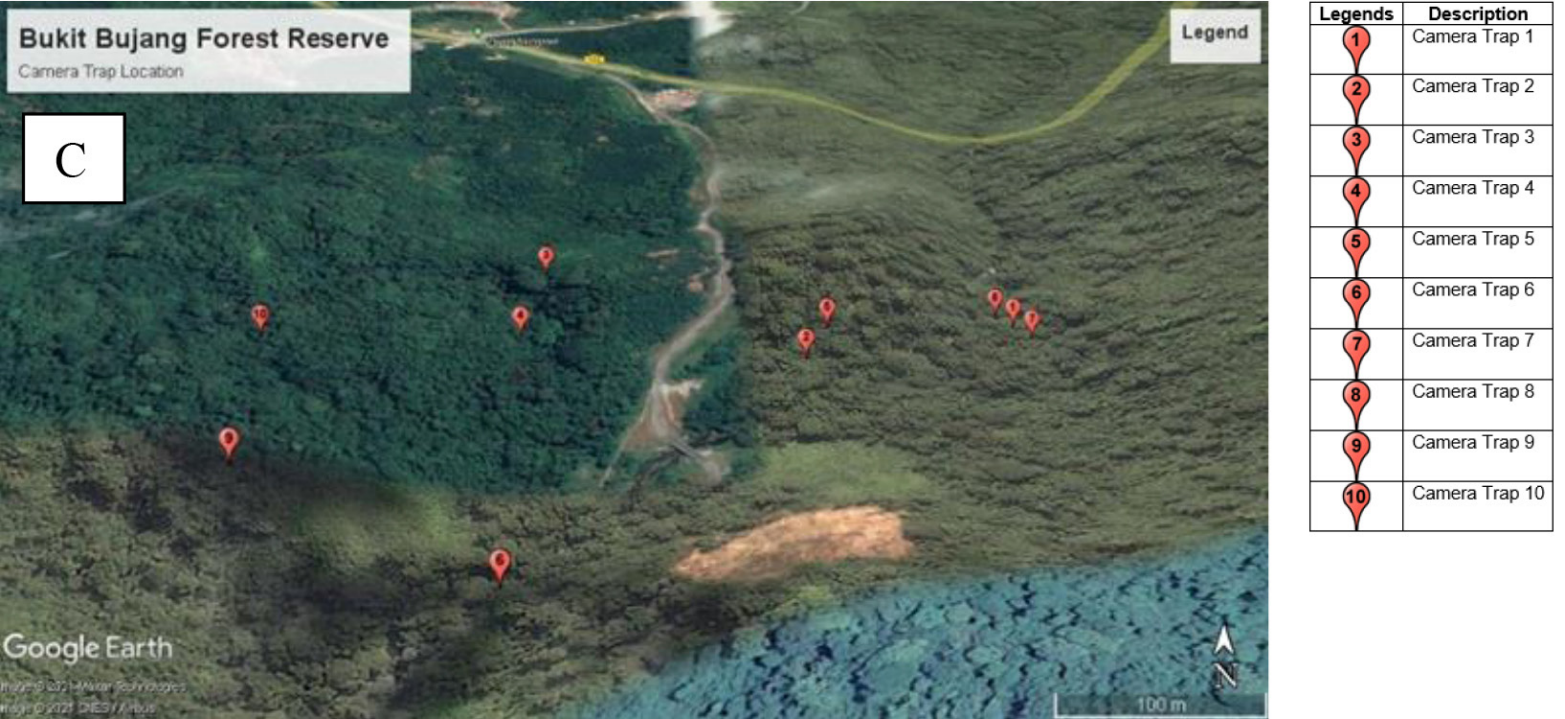
Distribution of duplicated 10 camera traps in each study sites. (A) Terla FR, (B) Bertam FR and (C) Bukit Bujang FR (Adapted from Google Earth Pro V 7.1).

Camera traps were deployed at random locations in each study sites (Fig. 3) depending on the ecological condition of the area to avoid biases in the data collected of non-volant small mammals that passed through the camera (Francesco *et al.* 2010). Each camera deployment point was chosen based on the presence of visible animal trails, footprints, scents, activity areas (e.g., big wallows left by Eurasian wild pig [Azhar *et al*. 2016]) and tree marks left by wildlife (e.g., scratching marks of sun bear on tree trunks [Sasidhran *et al.* 2016]), near streams or in the vicinity of streams (Azhar *et al.* 2016). Baits were not used in this study to avoid any specific preferences or bias in surveying medium (>1 kg) and large-sized non-volant small mammal species (Tee *et al.* 2018).

All 10 units of camera traps were left for five consecutive days for each site per sampling time. The total sampling days for camera trap method were 100 (10 traps × 5 days × 2 phases) within two phases of sampling time. Number of each species photo-captured by random cameras were recorded to calculate species richness.

### Data Analysis

The species diversity indices, Shannon-Wiener Index (*H′*), Evenness (*E*) and Dominance (*D*) were used to calculate the species richness of each selected localities using the Paleontological Statistics (PAST) software (Hammer *et al.* 2001). Shannon Weiner index (Shafie *et al.* 2014; Estrada-Villegas *et al.* 2012; Dee *et al.* 2019) was used to analyse the diversity index of non-volant small mammals in the four main habitats of restoration, boundary, disturbed forest and undisturbed forest area.

According to Mishra, Pandey, *et al.* (2019), One-way ANOVA is a statistical technique extended from an independent t-test to compare the mean for more than three groups of an independent variable. Therefore, one-way ANOVA was used to analyse the influence of different habitats on the number of individuals. Moreover, all the variables are considered as significantly different at *p* < 0.05 (Mishra, Singh, *et al.* 2019). The trapping frequency of each species was calculated by dividing the total number of species captures by the total number of all captures (Ruppert *et al.* 2021).

## RESULTS AND DISCUSSION

### Recorded Species of Non-volant Small Mammals in Cameron Highlands

A total of 23 species of non-volant small mammals from six orders and nine families were recorded by live trapping and camera trapping at the three study sites of Terla A, Bertam and Bukit Bujang Forest Reserve. All the recorded non-volant small mammals were captured in four habitat types: restoration, boundary, disturbed forest and undisturbed forest area.

Based on Table 1, there were 11 species from the family Muridae (*Berylmys bowersi*, *Leopoldamys sabanus*, *Maxomys whiteheadi*, *Niviventer cameroni*, *N. cremoriventer*, *N. fulvescens*, *Rattus exulans, R*. *norvegicus*, *R. tanezumi*, *R. tiomanicus* and *Sundamys muelleri)*; one (1) species from the family Erinacidae (*Hylomys suillus*), Felidae (*Prionailurus bengalensis*), Herpestidae (*Urva urva*), Manidae (*Manis javanica*), Tupaiidae (*Tupaia glis*), Tragulidae (*Tragulus kanchil*); three (3) species from the family Sciuridae (*Callosciurus caniceps*, *Dremomys rufigenis* and *Lariscus insignis)*; and three (3) species from the family Viverridae (*Paguma larvata*, *Paradoxurus musangus* and *Prionodon linsang)*.

In terms of conservation status (Table 1), all non-volant small mammals recorded were classified as Least Concern (LC) in the IUCN (2021) except for one species belonging to the family Manidae (*Manis javanica*) which is classified as Critically Endangered (CR). Two species from the family Muridae, namely *Maxomys whiteheadi* and *Niviventer cameroni* is classified as Vulnerable (VU). The two rodent species, *N. cameroni* and *M. whiteheadi* are generally regarded as crop pests and could be subjected to population control measures by human habitation in the rural areas. *Niviventer cameroni* is a montane species, endemic to the Cameron Highlands of peninsular Malaysia.

Most of the non-volant small mammals captured are classified as Least Concern (LC) by PERHILITAN (2017) except for *Hylomys suillus* and *Berylmys bowersi* which are Data Decifient (DD). Under WCA (2010), *Tragulus kanchil* and *Paradoxurus musangus* are listed as Protected (P), *Hylomys suillus*, *Prionailurus bengalensis*, *Urva urva*, *Manis javanica*, *Niviventer fulvescen*, *Rattus exulans*, *Tupaia glis*, *Paguma larvata* and *Prionodon linsang* as Totally Protected (TP), while the rest are listed as Not Protected (NP).

### Species Richness

Surveys at the three localities at Terla A, Bertam and Bukit Bujang Forest Reserve successfully recorded a total of 23 species representing nine families, from which 17 species were captured by live traps and 12 species from camera trap photos or videos (Table 1). The family Muridae recorded the highest number of non-volant small mammals captured. Almost 47.83% of the individuals captured fall in this order of Muridae with 11 species recorded using live traps and three species recorded using camera traps. Musser (2017) attributed high population of rodents to the availability of food and shelter as they co-exist with humans. On the other hand, the family Muridae showed that it is relatively common in all three study sites, with no significant difference in numbers between the study sites. Small mammals, particularly non-volant small mammals, have a distinct habitat specialisation and can be classified as forest and open land specialists and habitat generalists, each responding differently to changes in landscape complexity (Gentili *et al.* 2014). Both Sciuridae and Viverridae recorded only three species from each family (13.04%).

The other six families, namely Erinacidae, Felidae, Herpestidae, Tragulidae, Tupaiidae and Manidae were represented by a single specimen, each only representing 4.34% of all captures. These six families of non-volant small mammals could be rare, as only single individuals were captured for the entire study period.

### Live Trap Method

**Figure 4:**
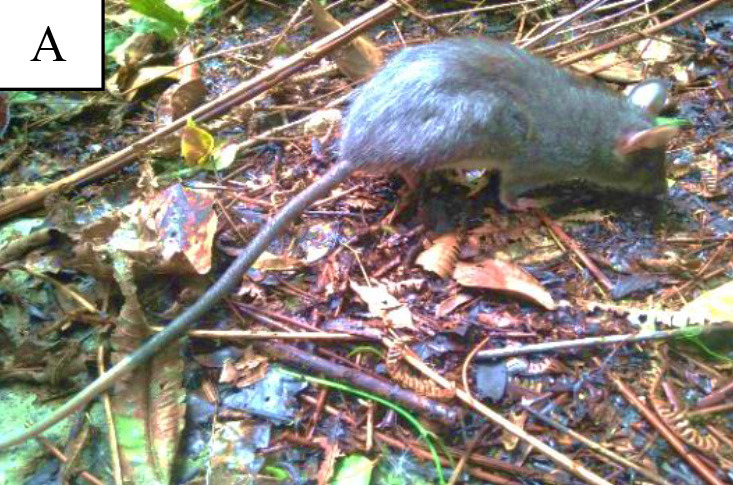

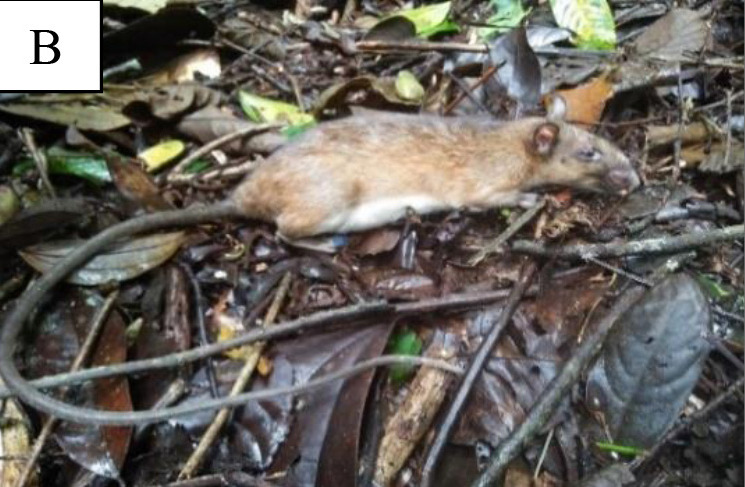

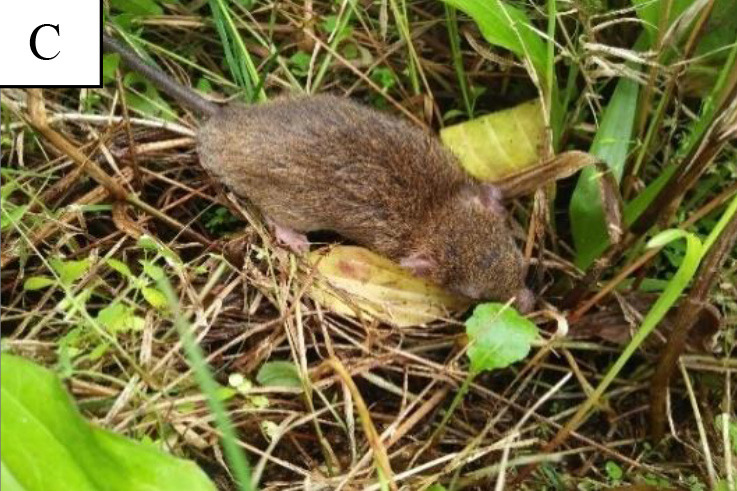

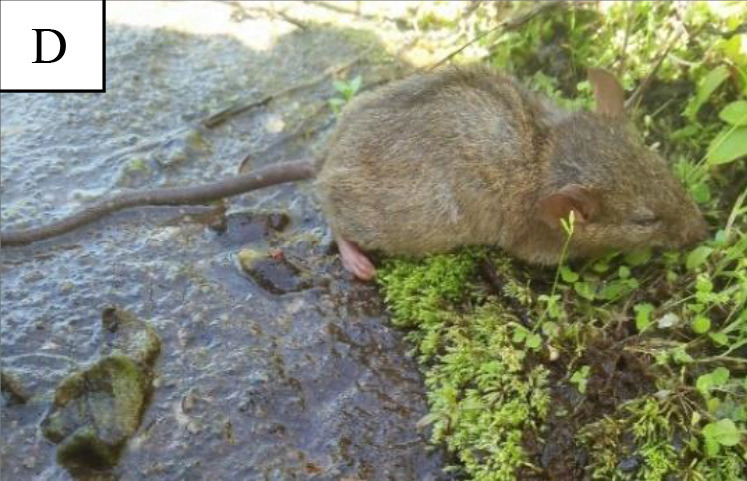

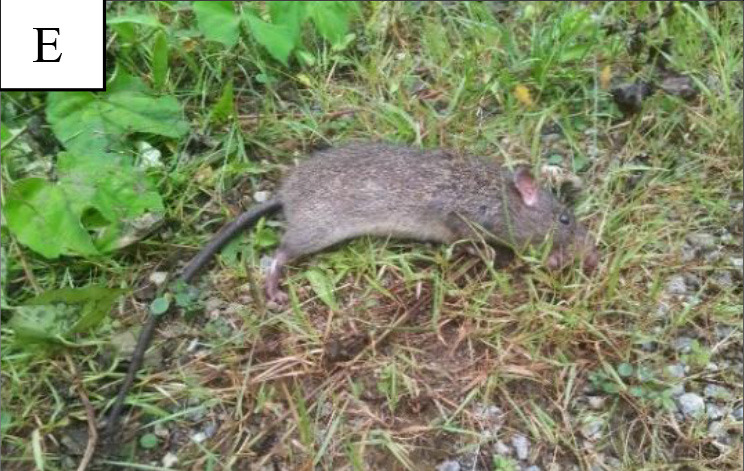

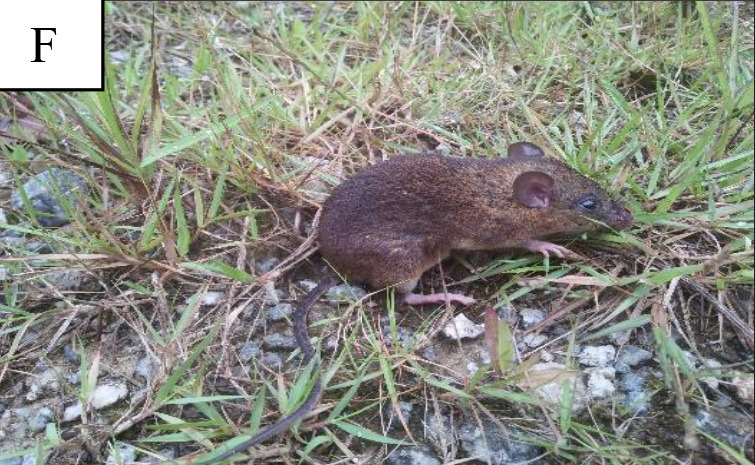

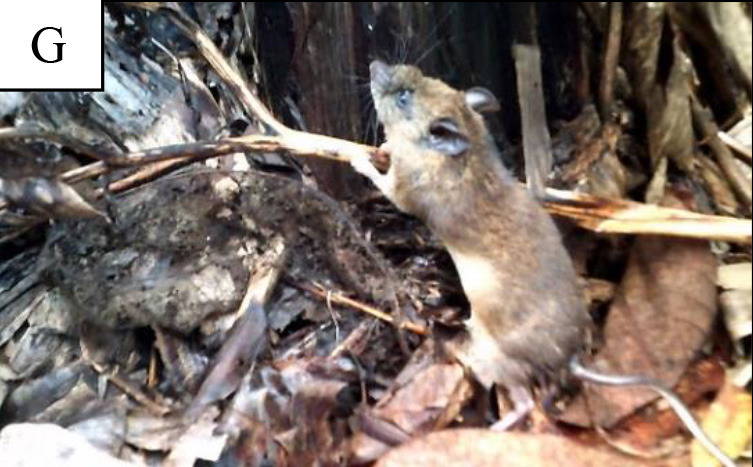

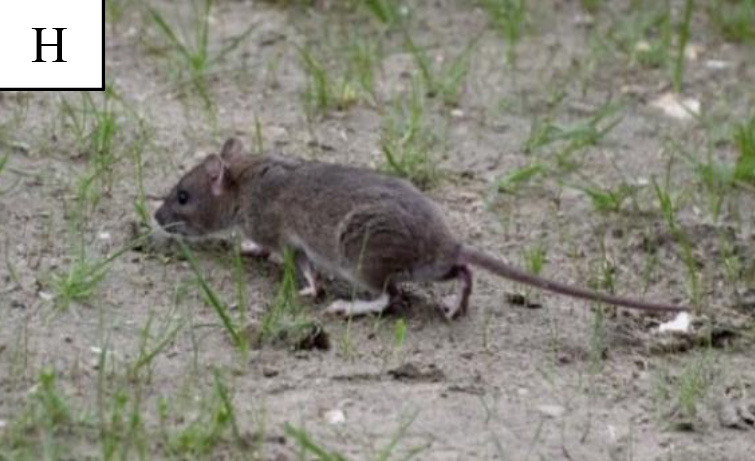

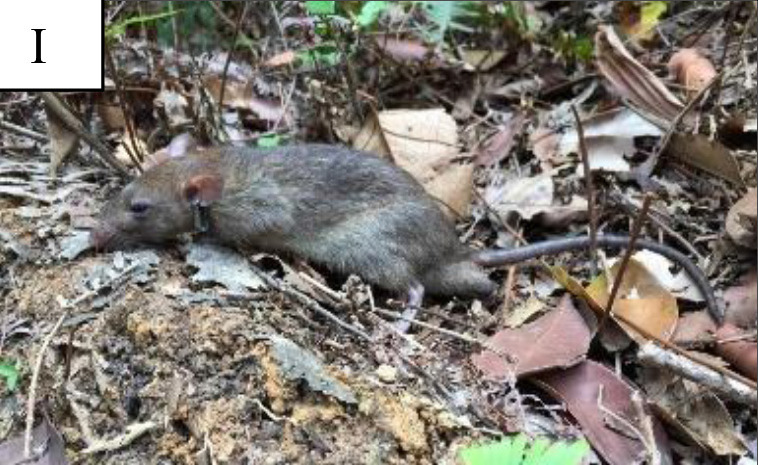

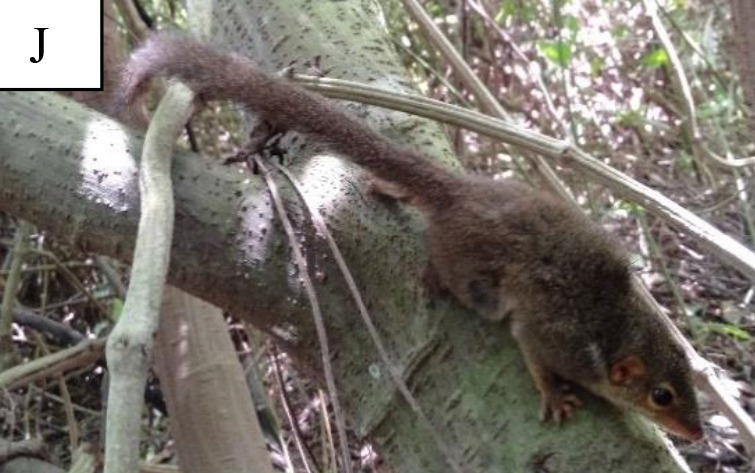

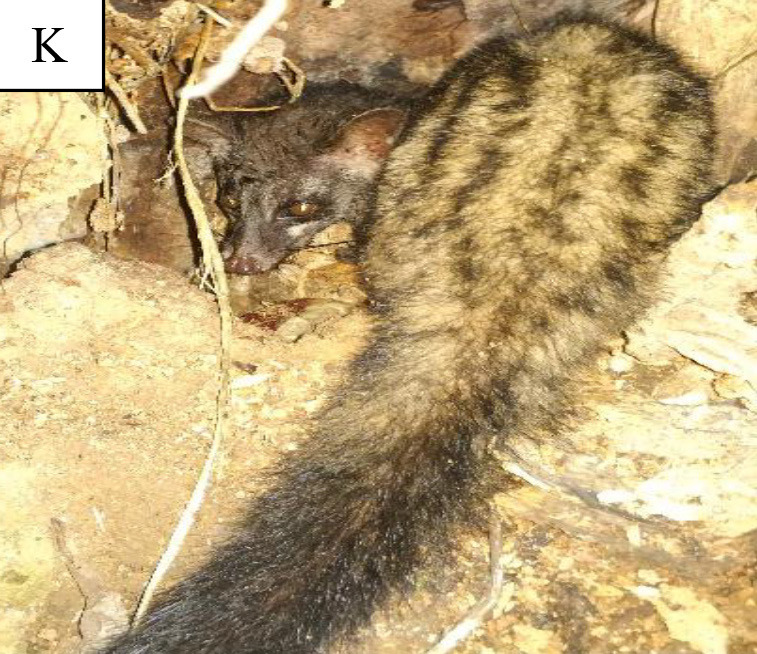

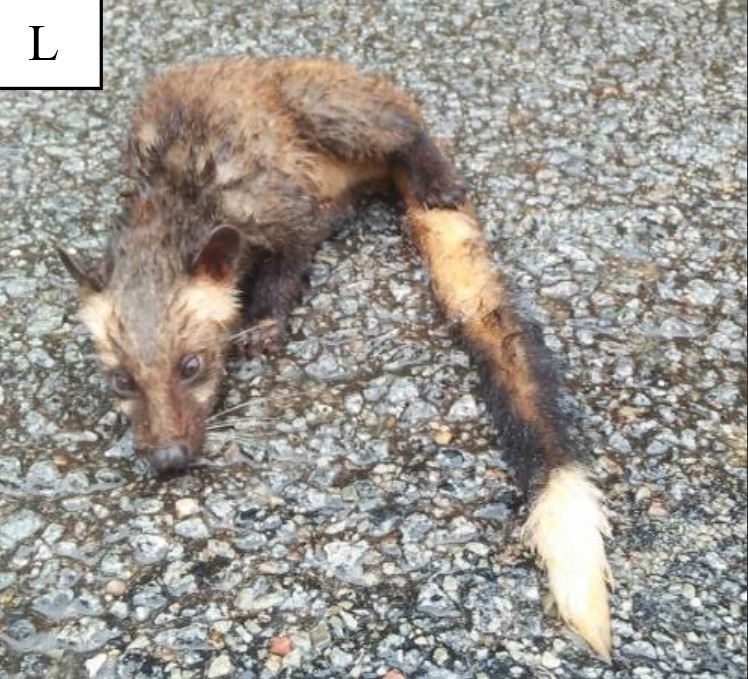

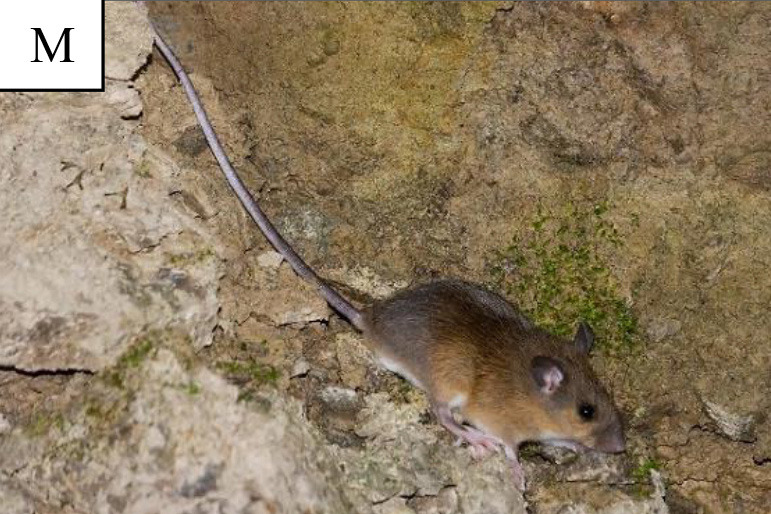

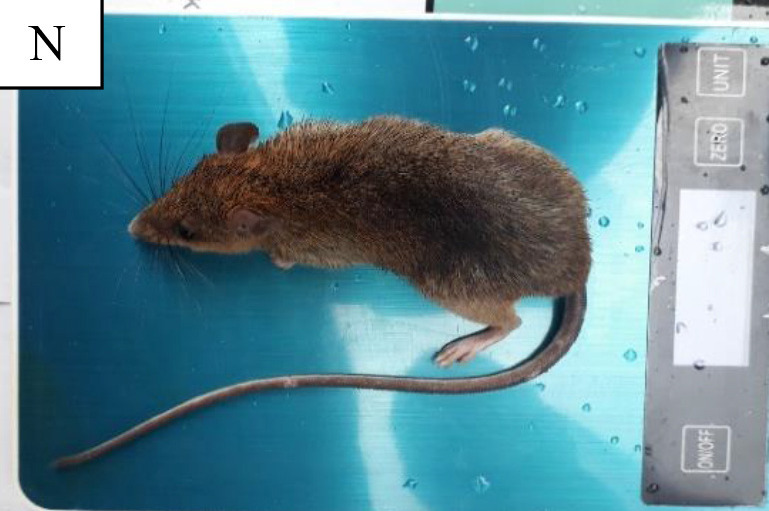

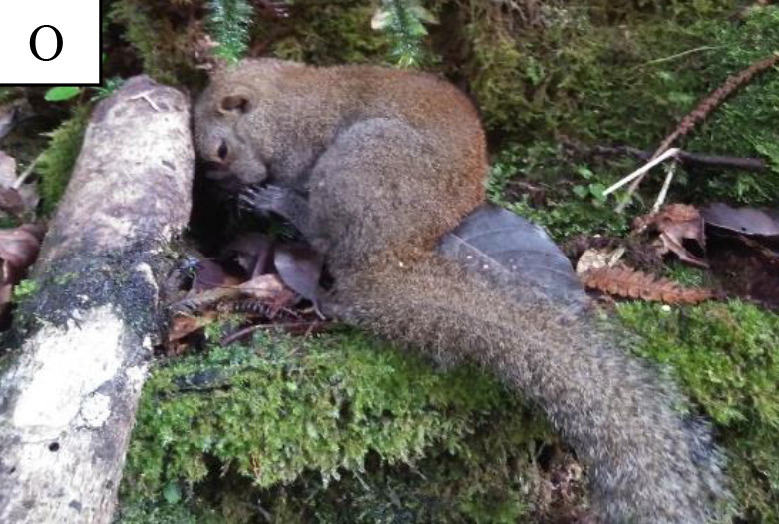

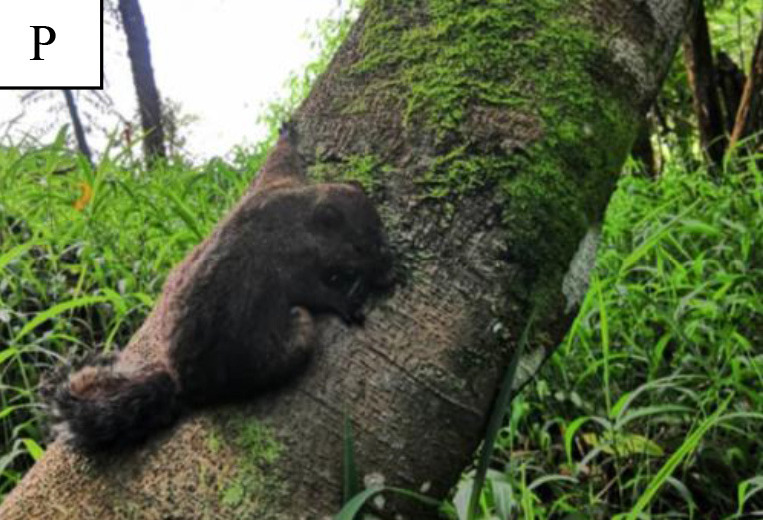

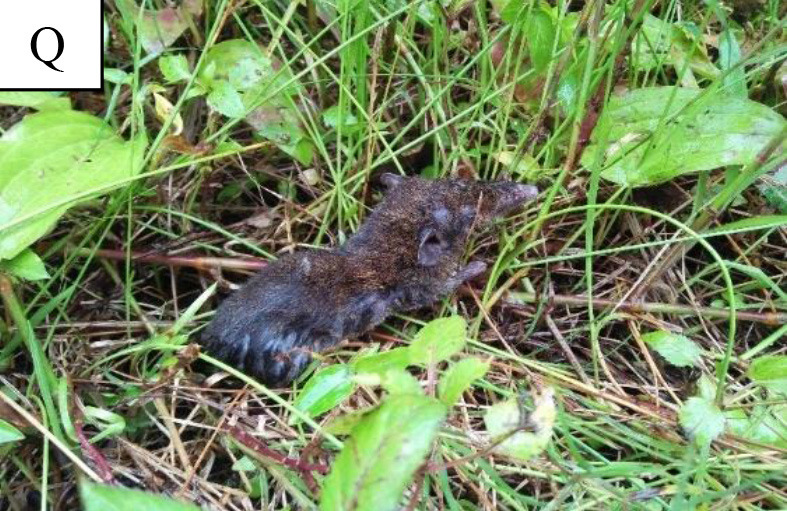
17 species caught by live trapping in Cameron Highlands, Pahang. (A) Berylmys (*B. bowersi*); (B) Leopoldamys (*L. sabanus*); (C) Rattus (R. exulans); (D) Rattus (*R. Tiomanicus*); (E) Mullers (*S. muelleri*); (F) Maxomys (*M. whiteheadi*); (G) Niviventer (*N. cremoriventer*); (H) Rattus (*R. norvegicus*); (I) Rattus (*R. tanezumi*); (J) Treeshrew (*T. glis*); (K) Common Palm Civet (*P. musangus*); (L) Masked Palm Civet (*P. larvata*); (M) Niviventer (*N. fulvescens*); (N) Niviventer (*N. cameroni*); (O) Callosciurus (*C. caniceps*); (P) Red-cheeked Squirrel (*D. rufigenis*); (Q) Gymnure (*H. suillus*). Not to scale.

Live trapping yielded 39 individuals of non-volant small mammals from five families. Overall, there were Viverridae (2 species), Erinacidae (1 species), Muridae (11 species), Sciuridae (2 species) and Tupaiidae (1 species) (Fig. 4) (Table 2).

Muridae was the most common family with 11 species caught, *Berylmys bowersi* being the species with highest number of individuals caught (seven) and the species mostly frequently caught close to forest litter. *Sundamys muelleri* came in second with six individuals and 15.38% of the captures. *Sundamys muelleri* has a wide distribution and was normally caught near rivers (Francis & Barrett 2008; Jayaraj *et al.* 2012). Additionally, Jayaraj *et al.* (2012) reported this big rodent was caught in the limestone area of Gua Ikan where there is a river flowing into the cave. In Borneo, this species was caught on ground or on low trees (Wilson *et al.* 2006). Zakaria *et al.* (2001) also reported that this species can survive in disturbed habitats.

Ten species, i.e., *Paguma larvata*, *Paradoxurus musangus*, *Hylomys suilus*, *Niviventer cameroni*, *N. fulvescen*, *Rattus tanezumi*, *R. exulans*, *R. tiomanicus*, *R. norvegicus*, *Tupaia glis* and *Callosciurus caniceps* were found in the trapping exercise with one individual representing each species. In conjunction with the study, two species from the family Viverridae i.e *Paradoxurus musangus* and *Paguma larvata* were caught in the traps as they are common in both pristine and disturbed forests. Both species can adapt in primary and secondary forests, albeit lower in the latter than the former (Nakashima *et al.* 2010). This could possibly explain the capture of single individuals of both species in the study sites.

*Paradoxurus musangus* has a wide geographical distribution and global presence due to its adaptability to a wide range of habitats (Duckworth *et al.* 2016). In Bukit Timah Nature Reserve (BTNR) Singapore, the presence of feral populations that were originally pets deliberately released by or escaped from their owners contributed to the increase in illegal poaching of the species (Chan & Davison 2019).

The adaptation of *M. whiteheadi* in the undisturbed area (Table 2) shows that this habitat type is preferred by this species (Chapman *et al.* 2019). According to Francis and Barrett (2008), this species can be found in tall and undisturbed secondary forests, occasionally encroaching disturbed areas in the vicinity of these forests.

### Diversity indices and Relative Abundance for Live Trap Method

Species diversity, Shannon Wiener (*H′*) was higher at Terla A Forest Reserve (*H′* = 2.274) compared to Bertam and Bukit Bujang Forest Reserve. In contrast, the species evenness (*E*) in Bertam (*E* = 0.857) was lower than Bukit Bujang Forest Reserve (*E* = 0.931). The value of Simpson’s Dominance Index, *D*, indicated a low species dominance at Bertam at 0.806 while the value of species dominance is slightly higher in Bukit Bujang FR at 0.809 (Table 3). According to Rabosky (2009), different ecological limits seem to be the main determinants of diversification and, therefore, species richness. The *Chao-1* estimator indicated that Terla A was the richest area. The results of this study revealed that a recovered land of the secondary forest had an impact on the diversity and distribution of non-volant small mammals. The age of the restoration area contributes to the growth of vegetation in each habitat (Derhe *et al.* 2017). The restoration project for Terla A and Bertam Forest Reserves began in November 2017 and no restoration projects were ongoing in Bukit Bujang (Forestry Department of Peninsular Malaysia 2020). Although secondary and recovering forests may harbor a similar number of species as mature forests (Poorter *et al*. 2021), communities in secondary forests are usually dominated by generalist species (Gardner *et al.* 2007). According to Whitehead *et al.* (2014), restoration sites were progressing towards becoming a rainforest and deviating from pasture sites in their small–medium mammal composition.

Table 4 shows the species diversity, Shannon Wiener (*H′*) in the boundary area (*S* = 8, *H′* = 2.025) and disturbed forest area (*S* = 8, *H′* = 1.992) shows the same total of number of species (*S*) compared to other study habitats; restoration area has the lowest species diversity (*S* = 3, *H′* = 0.950). In contrast, the species evenness (*E*) in the disturbed forest area (*E* = 0.916) is lower than in the undisturbed forest area (*E* = 0.930). The value of Simpson’s Dominance Index, *D*, shows low species dominance in restoration area at 0.56 while the value of species dominance is slightly higher in the disturbed forest area at 0.86. The *Chao-1* species richness estimator indicated that the boundary area was the richest area. The restoration habitat contributed to the least number of non-volant small mammals compared to other habitats, likely because the habitat was quite open without tree canopies compared to other habitats. According to Yaap *et al.* (2010), the presence of non-volant small mammals in this particular habitat was influenced by the availability of feed, water, and shelter provided by the nearby settlement areas that surrounds the forest. Zakaria *et al.* (2001) also stated that rodents can sustain themselves with seeds, fruits and plant matter from the natural vegetation without any detrimental effects on the habitat/ecosystem.

Cold weather and limited food sources such as fruit could be possible factors that lead to low diversity in mountain habitats (Shukor 1997; Butler & Lawrence 2019). Non-volant small mammals such as rodents and insectivores are highly mobile animals whose distribution is influenced by the altitude and vegetation types as well as human disturbance (Mulungu *et al.* 2008; Sukma *et al.* 2019).

Although the Evenness (*E*) at Bukit Bujang FR recorded the lowest values for both abundance and diversity compared to the other two sites, the differences between all three study areas were found to be significant. Such a result in Bukit Bujang FR may be affected by the habitat itself, where the relative abundance, in reference to in Pardini *et al.* (2005) and Cabrini *et al.* (2013), may be more negatively sensitive to forest fragmentation and isolation than to species richness.

One-way ANOVA analysis performed between sites, there were significant differences in non-volant small mammals’ diversity for Terla A, Bertam and Bukit Bujang Forest Reserve (F (5,22) = 3.086, *p* = 0.029). Contrasting vegetation and intensities of anthropogenic activities found at each site may explain the observed variations. In previous studies, Ramli and Hashim (2009) have reported that small mammal populations in tropical forests have seasonal variations and variations in population structure, density, biomass and species richness even if they live within the same region but in different habitat types.

### Camera Trapping

The total field effort comprised of 10 camera traps, over a cumulative period of five days for each site, recorded a total of 94 photographs. A total of 12 species from eight families were recorded from three study sites where Bertam FR captured the most photographs with a total of 46 photos (Table 5). Forty squirrels, *Lariscus insignis* and *Callosciurus canicep* recorded the highest photos captured. A Sciurids, *L. insignis* which is considered to be elusive and typically trap shy as it has never been live trapped to date, was successfully recorded by the camera trap. Saiful *et al.* (2001) reported the home range of *L. insignis* as too small which posed a challenge for live trapping as only one individual was successfully trapped in their study in Ulu Gombak. Ruppert *et al.* (2021) also encountered only one individual of *Lariscus insignis* in their study in Penang Island.

One photo of each species including *Prionailurus bengalensis* (Leopard Cat), *Manis javanica* (Sunda Pangolin), *Paguma larvata* (Masked Palm Civet) and *Prionodon linsang* (Banded Linsang) was captured. The presence of listed species in Bukit Bujang FR, especially *M. javanica* (Sunda Pangolin) in undisturbed forest area has been classified as Critically Endangered due to poaching and international illegal trade (Phillipps & Phillipps 2016; IUCN 2021).

In reference to the species inventory of Cameron Highlands, *Tragulus kanchil* is a newly recorded species in Cameron Highlands. Based on camera trap results, *T. kanchil* develops nocturnal pattern behaviour as they became elusive due to fragmentation and frequent encroachment. According to Farida *et al.* (2006), *Tragulus* sp. is typically shy and rarely seen in the forest and has only been caught in camera trap foraging the forest floor looking for fruits.

In the present study, camera trapping techniques were unable to identify non-volant small mammal species such as rats and squirrels, presumably, due to their small size but can be classified under the order of Rodentia such as *Rattus* sp. and *Sciurus* sp. According to Nick *et al.* (2021), their size is often insufficient to trigger infra-red sensors, and resultant images may be of inadequate quality for species identification with a possibility that some species not being able to be identified.

Camera trapping eliminates the need to handle an individual physically. It offers a method for detecting rare, elusive, or trap-shy individuals that may be missed by traditional, intensive, shorter-duration live trapping methods (Gray *et al.* 2017; Rendall *et al.* 2014). According to Pollock *et al*. (2002), the major limitation associated with the use of camera traps for terrestrial mammals is that some species may not be detected.

### Camera Trap Recording Species

**Figure 5:**
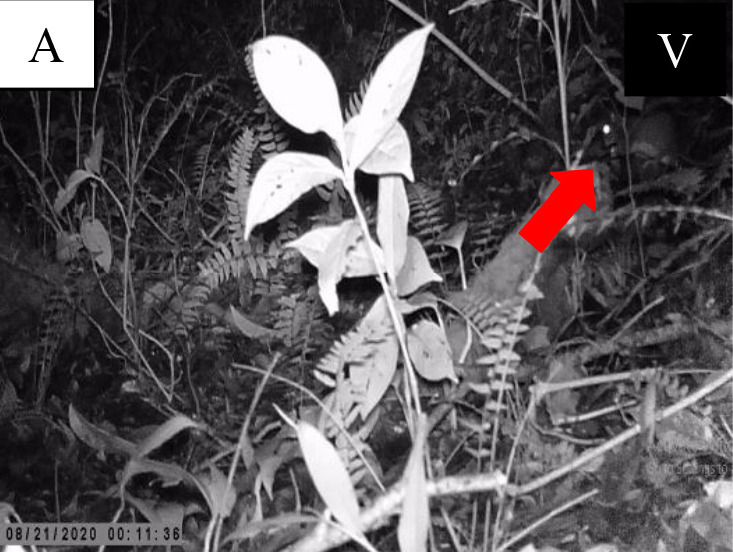

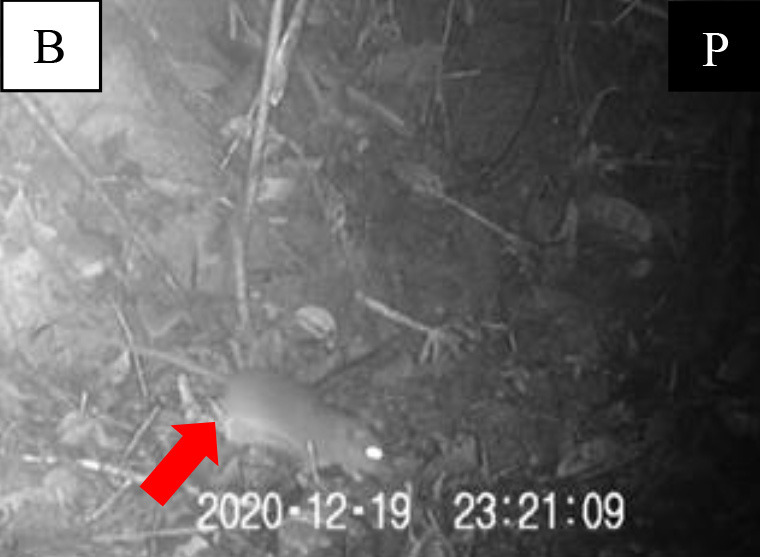

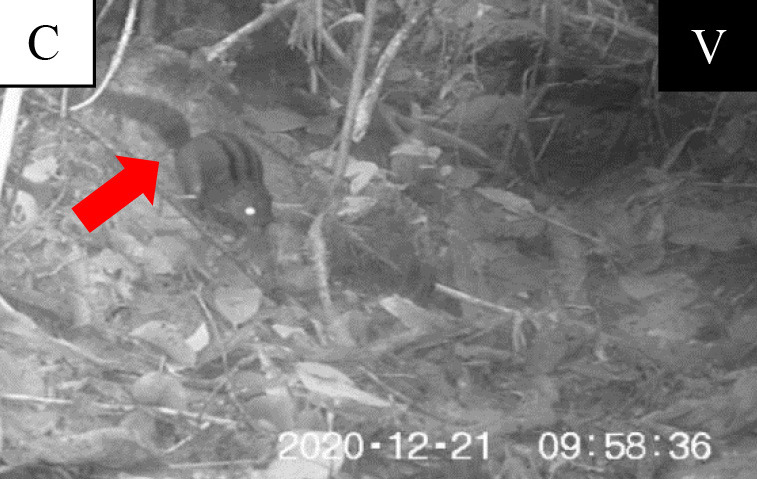

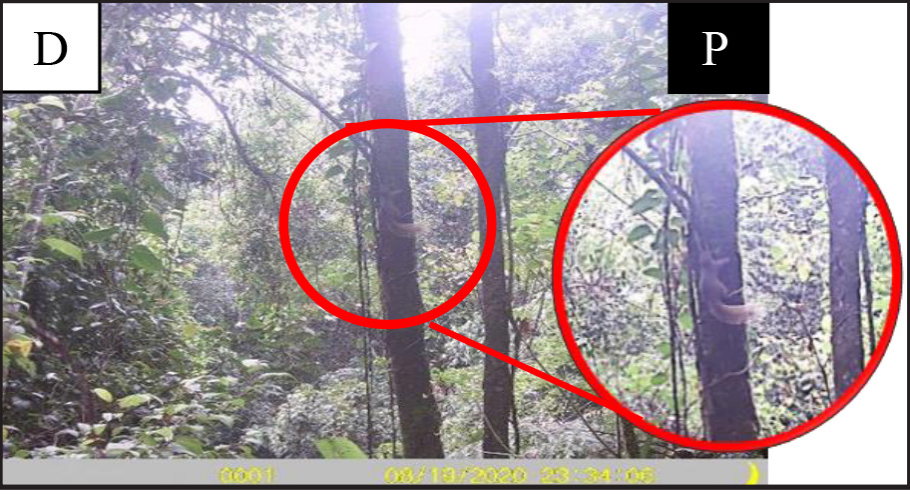
Record of four rodent species from Sciuridae family. (A) *Berylmys bowersi*; (B) *Leopoldamys sabanus*; (C) *Lariscus insignis*; (D) *Callosciurus caniceps*. *Note*: V = video; P = photo.

**Figure 6:**
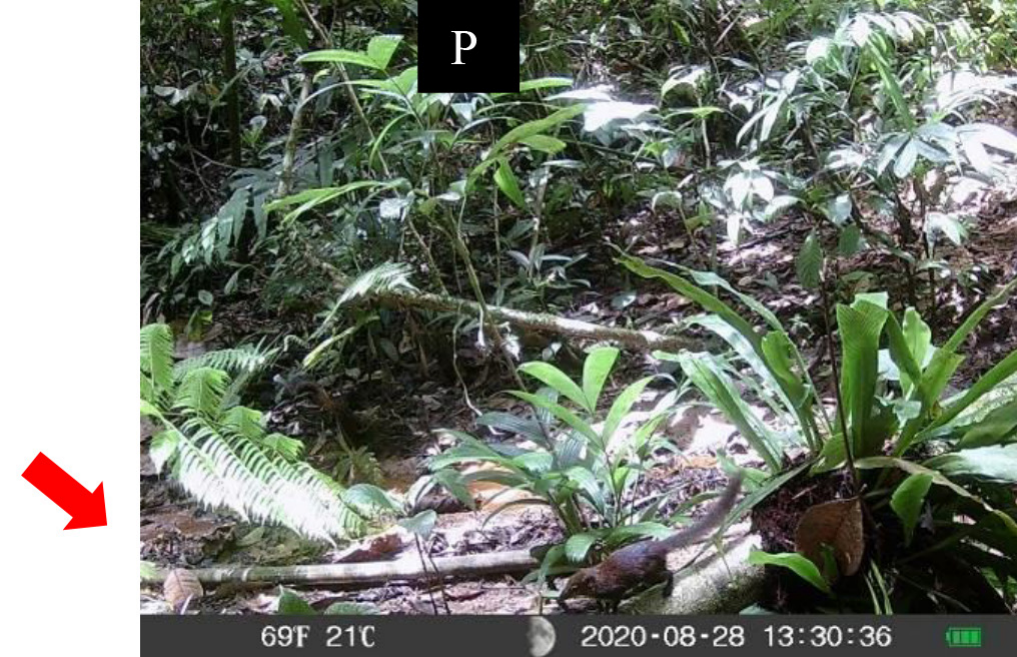
Record of *Tupaia glis* from Tupaiadae family. *Note*: P = Photo.

**Figure 7:**
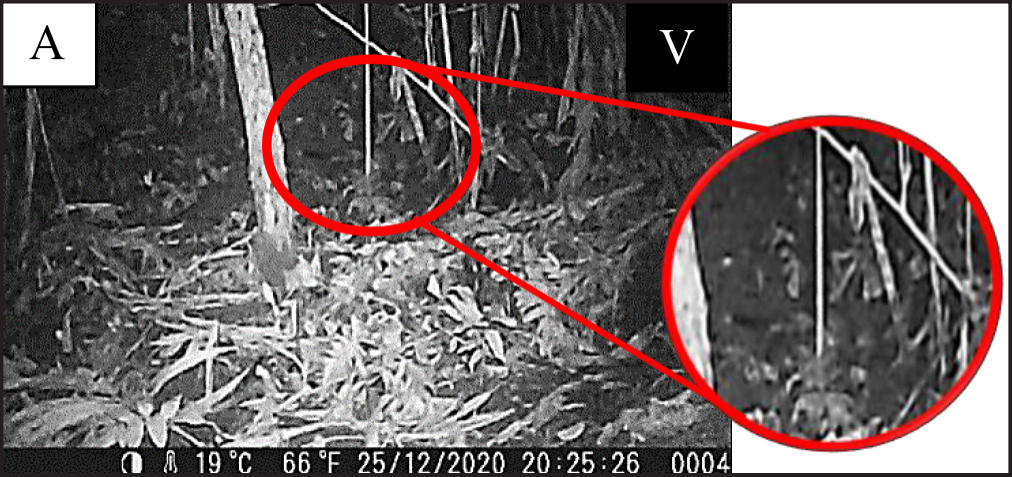

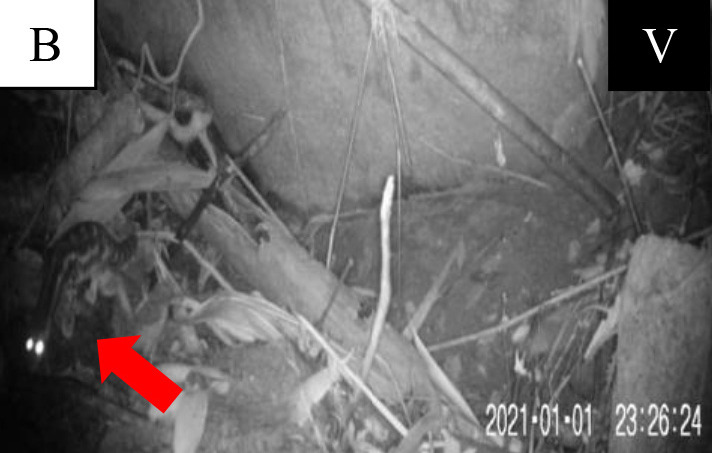
Record of two species from Viverridae family. (A) *Paguma larvata*; (B) *Prionodon linsang*). Note: V = video; P = photo

**Figure 8:**
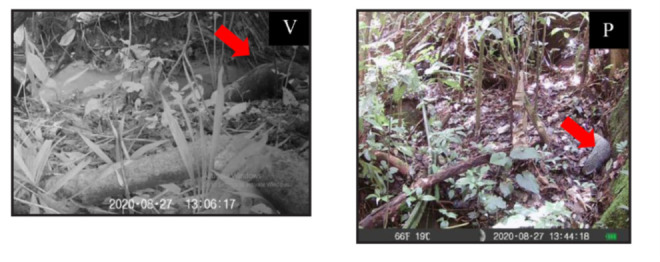
Record of *Urva urva* species from Herpestidae family. Note: V = video; P = photo

**Figure 9:**
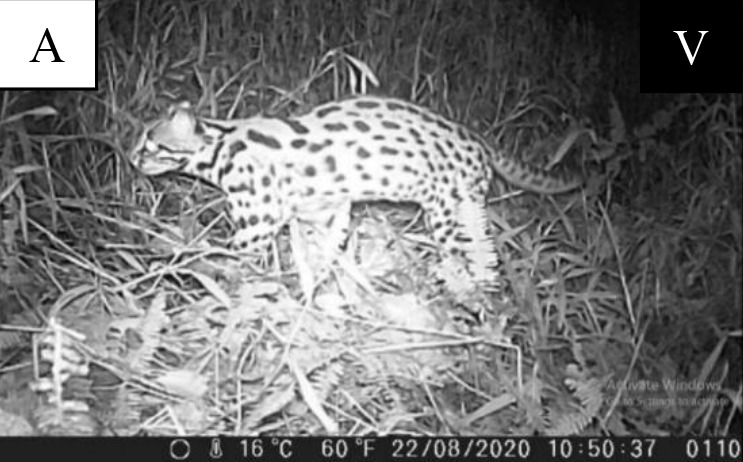

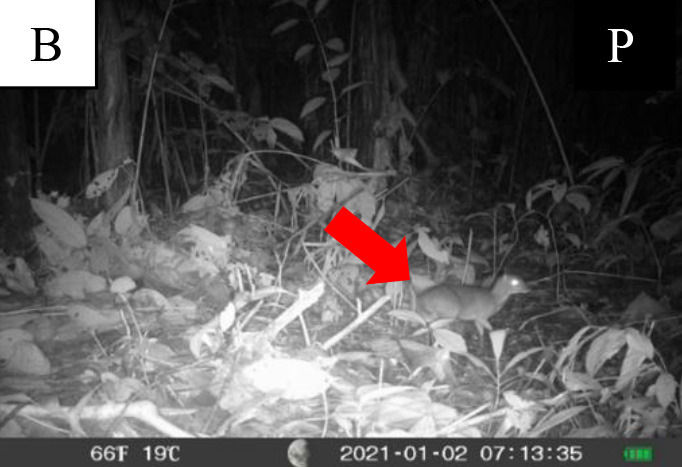
Record of (A) Felidae: *Prionailurus bengalensis* (Leopard cat); (B) Tragulidae: *Tragulus kanchil.* Note: V = video; P = photo

Camera trap records consists of 94 photos and videos of non-volant small mammals recorded at three forest reserves (Figs. 5–9). From 94 photos, 12 species from eight families were successfully documented at Terla A FR, Bertam FR and Bukit Bujang FR in Cameron Highland.

### Comparison of The Present and Previous Studies on Non-Volant Small Mammals in Cameron Highlands

Recent study recorded 17 species of non-volant small mammals captured by live traps and 12 species from camera trap photos. Previous study conducted by Shahfiz *et al.* (2011) in two forest reserves in Cameron Highlands recorded a total of 18 individuals from five species of non-volant small mammals captured in Mentigi and Ulu Bertam Forest Reserve. The common treeshrew (*Tupaia glis*), grey-bellied squirrel (*Callosciurus caniceps*) and white-bellied rat (*Niviventer fulvescens*) were caught in the study where five individuals were recorded for each species.

Studies conducted by Shahfiz *et al.* (2011) and Nur Laila (2019) are in parallel with current study where they also found *Berylmys bowersi*, *Niviventer cremoriventer* and *Tupaia glis* in Cameron Highland. Nine individuals from four species in Terla A Forest Reserve, Cameron Highlands comprising of Muridae, Sciuridae and Tupaiidae family were captured by Nur Laila (2019). The most abundant species were *N. cremoriventer* (four individuals) followed by *Dremomys rufigenis* (three individuals) and current study in Terla A Forest Reserve also recorded three individuals of *Dremomys rufigenis.* However, only one individual *T*. *glis* was captured using live trap and caught at the boundary area in Terla A Forest Reserve. *Tupaia glis* can be easily observed foraging and moving around in the presence of human (Noor Aisyah *et al*. 2016) as they have high tolerance to habitat disturbance (Nur Syazana *et al*. 2013; Muhammad Hafiz *et al.* 2015).

## CONCLUSION

Twenty-three non-volant small mammal species were recorded in this study. Overall, Terla A Forest Reserved recorded the highest diversity of non-volant small mammals with 14 species. Although the restoration habitat differed greatly from the boundary, disturbed and undisturbed forest area in terms of species richness, it still appears to have an important role in providing habitat for highly adaptable species. Therefore, it is crucial for the authorities to manage these non-protected areas properly as they continue to function as an ecosystem. In addition, some species are endemic such as *N. cameroni* and *N. cremoriventer* to specific habitats or elevations (Pimsai *et al.* 2014), making them a high conservation priority.

One limitation is that although overall species diversity of small non-volant mammals is high, trap success may have been low in some parts of the habitat due to weather conditions during data collection. It was raining during the sampling period which may have affected the activity of non-volant small mammals. This could have impacted our data because the mammals would have been less likely to roam around. Therefore, many species may not have been detected in the rapid survey.

## Figures and Tables

**Table 1:** Conservation status of non-volant small mammals recorded at Terla A, Bertam and Bukit Bujang Forest Reserve.

Family/Species	Common name	IUCN (2021)	PERHILITAN (2017)	WCA (2010)	Percentage (%)	Capture method
Erinacidae						
*Hylomys suillus* (Müller, 1840)	Short-tailed gymnure	LC	DD	TP	4.34	LT
Felidae						
*Prionailurus bengalensis* (Kerr, 1792)	Leopard cat	LC	NT	TP	4.34	CT
Herpestidae						
*Urva urva* (Hodgson, 1836)	Crab-eating mongoose	LC	NT	TP	4.34	CT
Manidae						
*Manis javanica (*Desmarest, 1822)	Sunda pangolin	CR	CR	TP	4.34	CT
Muridae						
*Berylmys bowersi* (Anderson, 1879)	Bower’s rat	LC	DD	NP	47.83	LT, CT
*Leopoldamys sabanus* (Thomas, 1887)	Long-tailed giant rat	LC	LC	NP		LT, CT
*Maxomys whiteheadi* (Thomas, 1894)	Whitehead’s maxomys	VU	LC	NP		LT
*Niviventer cameroni* (Chasen, 1940)	Cameron Highlands niviventer	VU	LC	-		LT
*Niviventer cremoriventer* (Miller, 1900)	Dark-tailed niviventer	LC	LC	NP		LT
*Niviventer fulvescens* (Gray, 1847)	Indomalayan niviventer	LC	VU	TP		LT
*Rattus exulans* (Peale, 1848)	Pacific rat	LC	LC	TP		LT
*Rattus norvegicus* (Berkenhout, 1769)	Norway rat	LC	LC	NP		LT
*Rattus tanezumi* (Linnaeus, 1758)	House rat	LC	LC	NP		LT
*Rattus tiomanicus* (Miller, 1900)	Malaysia wood rat	LC	LC	NP		LT
*Sundamys muelleri* (Jentink, 1879)	Muller’s rat	LC	LC	NP		LT
*Rattus* sp.	-	-	-	-	-	CT
Sciuridae						
*Callosciurus caniceps* (Gray, 1842)	Grey-bellied squirrel	LC	LC	NP	13.04	LT, CT
*Dremomys rufigenis* (Blanford, 1878)	Red-cheeked squirrel	LC	VU	NP		LT
*Lariscus insignis* (F. Cuvier, 1821)	Three-striped ground squirrel	LC	LC	NP		CT
*Sciurus* sp.	-	-	-	-		CT
Tupaiidae						
*Tupaia glis* (Diard, 1820)	Common treeshrew	LC	LC	TP	4.34	LT, CT
Tragulidae						
*Tragulus kanchil* (Raffles, 1821)	Lesser mousedeer	LC	LC	P	4.34	CT
Viverridae						
*Paguma larvata* (C. E. H. Smith, 1827)	Masked palm civet	LC	NT	TP	13.04	LT, CT
*Paradoxurus musangus* (Raffles, 1821)	Common palm civet	LC	LC	P		LT
*Prionodon linsang* (Hardwicke, 1821)	Banded linsang	LC	LC	TP		CT

*Note:* IUCN = International Union for Conservation of Nature; WCA = Wildlife Conservation Act; LC = Least Concern, NT = Near Threatened, VU = Vulnerable, EN = Endangered; CR= Critically Endangered; DD = Data Deficient; P = Protected; TP = Totally Protected; NP = Not Protected; - = not assessed; LT = Live Trap; CT = Camera Trap

**Table 2:** Species of non-volant small mammals recorded in this study by using live traps.

Family species	Common name	Terla A Forest Reserve			Bertam Forest Reserve			Bukit Bujang Forest Reserve	Relative abundance (%)

		Restoration	Boundary	Disturbed forest	Restoration	Boundary	Disturbed forest	Undisturbed forest	
***Insectivora***									
*Hylomys suillus*	Short-tailed gymnure	-	1	-	-	-	-	-	2.56
***Muridae***									
*Berylmys bowersi*	Bower’s Rrat	2	-	1	1	1	2	-	17.95
*Leopoldamys sabanus*	Long-tailed giant rat	-	-	-	-	-	1	2	7.69
*Maxomys whiteheadi*	Whitehead’s maxomys	-	1	-	-	1	-	3	12.83
*Niviventer cameroni*	Cameron Highlands niviventer	-	-	1	-	-	-	-	2.56
*Niviventer cremoriventer*	Dark-tailed niviventer	-	-	-	-	-	1	2	7.69
*Niviventer fulvescens*	Indomalayan niviventer	-	1	-	-	-	-	-	2.56
*Rattus exulans*	Pacific rat	-	1	-	-	-	-	-	2.56
*Rattus norvegicus*	Norway rat	-	-	-	-	-	-	1	2.56
*Rattus tanezumi*	House rat	1	-	-	-	-	-	-	2.56
*Rattus tiomanicus*	Malaysia wood rat	1	-	-	-	-	-	-	2.56
*Sundamys muelleri*	Muller’s rat	-	1	1	-	1	1	2	15.38
*Callosciurus caniceps*	Grey-bellied squirrel	-	-	1	-	-	-	-	2.56
*Dremomys rufigenis*	Red-cheeked squirrel	-	1	2	-	-	-	-	7.69
***Tupaiidae***									
*Tupaia glis*	Common treeshrew	-	-	-	-	1	-	-	2.56
***Viverridae***									
*Paguma larvata*	Masked palm civet	-	-	-	-	-	-	1	2.56
*Paradoxurus musangus*	Common palm civet	-	-	-	-	-	1	-	2.56

**Total individual recorded**		4	6	6	1	4	6	11	

**Table 3:** Species abundance, richness and diversity values estimated for each study locality by using live trap method.

Study sites	Taxa (*S*)	Individuals	Simpson Dominance Index (*D*)	Shanon (*H′*)	Evenness (*E*)	*Chao -1*
Terla A	11	16	0.883	2.274	0.883	25
Bertam	7	12	0.806	1.792	0.857	9
Bukit Bujang	6	11	0.80	1.720	0.931	6.25

**Table 4:** Species abundance, richness and diversity values estimated for each study habitat by using live trap method.

Study habitat	Taxa (*S*)	Individuals	Simpson Dominance Index (*D*)	Shannon (*H′*)	Evenness (*E*)	*Chao -1*
Restoration	3	5	0.56	0.950	0.862	4
Boundary	8	10	0.86	2.025	0.947	13
Disturbed Forest	8	13	0.852	1.992	0.916	9.5
Undisturbed Forest	6	11	0.809	1.72	0.931	6.25

**Table 5:** Species diversity and abundance of non-volant small mammals captured by camera traps.

Order	Family	Species	Local name	Numbers of photo (P)/video (V) captured			N

				Terla A	Bertam	Bukit Bujang	
Rodentia	Muridae	*Rattus* sp.	-	1(V)	12(P, V)	1(P)	14
		*Berylmys bowersi*	Bower’s rat	5(P/V)	-	-	5
		*Sundamys muelleri*	Muller’s rat	-	-	-	0
		*Leopoldamys sabanus*	Long-tailed giant rat	-	6(P, V)	2(V)	8
	Sciuridae	*Lariscus insignis*	Three-striped ground squirrel	3(P)	4(P, V)	9(P, V)	16
		*Callosciurus canicep*	Grey-bellied squirrel	9(P)	-	1	10
		*Sciurus* sp.	-	2(P)	12(P, V)	-	14
Scandetia	Tupaiidae	*Tupaia glis*	Common treehrew	-	3(P)	-	3
Carnivora	Viverridae	*Paguma larvata*	Masked palm civet	-	1(V)	-	1
		*Prionodon linsang*	Banded linsang	-	-	1(V)	1
	Herpestidae	*Urva urva*	Crab-eating mongoose	-	8(P)	-	8
	Felidae	*Prionailurus bengalensis*	Leopard cat	1(V)	-	-	1
Artiodactyla	Tragulidae	*Tragulus kanchil*	Lesser mousedeer	1(V)	0	11(P)	12
Pholidota	Manidae	*Manis javanica*	Sunda pangolin	-	-	1(V)	1

Total numbers of captured photos				22	46	26	94
